# Pathogenesis of West Nile Virus Lineage 2 in Domestic Geese after Experimental Infection

**DOI:** 10.3390/v14061319

**Published:** 2022-06-16

**Authors:** Hannah Reemtsma, Cora M. Holicki, Christine Fast, Felicitas Bergmann, Martin Eiden, Martin H. Groschup, Ute Ziegler

**Affiliations:** Friedrich-Loeffler-Institut, Federal Research Institute for Animal Health, Institute of Novel and Emerging Infectious Diseases, 17493 Greifswald-Insel Riems, Germany; hannah.reemtsma@fli.de (H.R.); cora.holicki@fli.de (C.M.H.); christine.fast@fli.de (C.F.); felicitas.bergmann@fli.de (F.B.); martin.eiden@fli.de (M.E.); martin.groschup@fli.de (M.H.G.)

**Keywords:** West Nile virus, lineage 2, Germany, geese, pathogenesis, experimental infection

## Abstract

West Nile virus (WNV) is an emerging infectious pathogen circulating between mosquitoes and birds but also infecting mammals. WNV has become autochthonous in Germany, causing striking mortality rates in avifauna and occasional diseases in humans and horses. We therefore wanted to assess the possible role of free-ranging poultry in the WNV transmission cycle and infected 15 goslings with WNV lineage 2 (German isolate). The geese were monitored daily and sampled regularly to determine viremia, viral shedding, and antibody development by molecular and serological methods. Geese were euthanized at various time points post-infection (pi). All infected geese developed variable degrees of viremia from day 1 to day 10 (maximum) and actively shed virus from days 2 to 7 post-infection. Depending on the time of death, the WN viral genome was detected in all examined tissue samples in at least one individual by RT-qPCR and viable virus was even re-isolated, except for in the liver. Pathomorphological lesions as well as immunohistochemically detectable viral antigens were found mainly in the brain. Furthermore, all of the geese seroconverted 6 days pi at the latest. In conclusion, geese are presumably not functioning as important amplifying hosts but are suitable sentinel animals for WNV surveillance.

## 1. Introduction

West Nile virus (WNV) is a mosquito-borne *Flavivirus* infecting the entire spectrum of vertebrates in almost all continents of the world, except for Antarctica [1]. WNVs circulate between mosquitoes as vectors (especially *Culex*) and birds as amplifying hosts with numerous species of birds reaching virus titers high enough to transmit the virus back to mosquitoes [2]. Highly susceptible bird species (for example *Passeriformes*, *Falconiformes*, and *Strigiformes*) can develop severe neurological problems that can lead to death. Mammals, such as horses and humans, are considered dead-end hosts due to the low viral loads in their blood [3]. Nevertheless, clinical infections can occur in humans and horses, with fatal outcomes in rare cases [1]. Therefore, it is important to assess the zoonotic potential emanating from domestic poultry.

Since its first discovery in a human in Uganda in 1937 [4], WNV has spread continuously and the incidence of sporadic infections in humans has increased steadily. Despite numerous outbreaks with neuroinvasive diseases in densely populated cities of Europe and the Middle East in the 1990s, the threat of the virus to human health was long underestimated [5]. Only from 1999 onwards did the virus come into public focus, with the first outbreak of WNV in North America. It occurred in New York and surrounding areas, with human encephalitis and a concurrent high mortality rate in crows and various exotic bird species from a local zoological garden [6]. The first recorded outbreak on a commercial farm (keeping exotic and domesticated ducks and geese) was described in the United States of America (USA) in 2005 [7]. This was followed by a high mortality rate and increased seroconversion of the birds from the affected farm [7]. Similar cases were noted by goose keepers in Canada, Israel, and Hungary [8].

Currently, all isolates of WNV are clustered into nine lineages [9], but so far only lineages 1 and 2 have been associated with human infections [10]. WNV lineage 1 includes WNV strains from Europe, the Middle East, Africa, East Asia, Australia, and North and Central America. Until its spread to Europe, WNV lineage 2 only included strains from Africa (for example, the first detected case in Uganda, as well as cases in sub-Saharan Africa and Madagascar) [11,12].

With the arrival of WNV lineage 2 in Europe in 2004, an upsurge in human cases was observed in Hungary, Greece, Austria, Italy, Romania, and Russia [12,13,14,15,16,17]. In 2018, a very strong increase in human cases (7.2-fold compared to 2017), together with an extensive geographic spread, was recorded [18]. This was probably due to the warm and humid spring with warmer than average summer temperatures in Europe [19,20].

Therefore, it was not surprising that a bird (the great grey owl, *Strix nebulosa*) infected with WNV lineage 2 (central European subclade) was found in Germany for the first time at the end of August 2018 [21]. By the end of 2018, a total of 12 cases in native wild and aviary birds and 2 cases in clinically affected horses in eastern and southeastern Germany were diagnosed [22]. Hibernation in native mosquitoes must have occurred [23] and WNV reemerged in July 2019. In 2019, a total of 76 cases in birds, 36 in horses, and 5 confirmed mosquito-borne, autochthonous human cases were discovered [21,24]. WNV hotspots are currently located in Saxony, Saxony-Anhalt, Berlin, and Brandenburg. In 2018 and 2019, single cases were also noted in Hamburg, Bavaria, and Mecklenburg–Western Pomerania [24]. In 2020 and 2021, WNV occurred in the same areas, with a slight trend towards Lower Saxony (in horses) and Thuringia (in birds) [25]. Phylogenetic studies indicated that WNV lineage 2 virus entry into Germany in 2018 must have occurred via Austria and the Czech Republic [21]. On the other hand, recently more detailed phylogeographic studies suggest that for 2018 and 2019 a total of six different entry events could have taken place [24].

Infectivity studies in the past have demonstrated the susceptibility of geese to the closely related WNV lineage 1 strains from New York (1999) [26] and Israel (1998) [27]. At present, it is important to know whether German commercial poultry are similarly susceptible to the less pathogenic WNV strains circulating in Europe. To this end, preliminary studies demonstrated that all three German domestic poultry species (chickens, ducks, and geese) could successfully be infected with a low-pathogenic WNV lineage 1 strain from Italy. The highest and longest-lasting viremia as well as virus excretion was detected in geese. Yet it was concluded that all three domestic species most likely do not act as a virus reservoir for the transmission of WNV lineage 1 via mosquitoes [28]. At that time, it was assumed that WNV would come to Germany from the south, and therefore in the form of WNV lineage 1 rather than 2 [28]. Differences in the pathogenicity of European WNV lineage 2 compared to lineage 1 strains have been described in a small number of experimental studies [29,30,31].

As WNV lineage 2 has been present in Germany since 2018, it is particularly important for the poultry industry to verify the susceptibility and pathogenesis of WNV lineage 2 to German poultry. Furthermore, from a public health point of view it is essential to know whether geese can act as amplifying hosts for the German WNV. Finally, it would be good to know whether geese, as free ranging poultry, could readily be used during the mosquito season as sentinel animals within the framework of a monitoring program.

For this purpose, domestic geese were experimentally inoculated with a German WNV strain from the 2018 outbreak via subcutaneous needle injection to imitate a mosquito bite. For the accurate tracking of the virus not only in blood or swab material but also in specific tissues at different time points post-infection (pi), three geese were euthanized and analyzed at interim time points (3, 6, 10, and 14 dpi). Days 3 and 6 pi were chosen because these coincided with the highest viral loads in the blood and swab samples in preceding experiments reported in the literature [27,28,32]. Day 10 was chosen due to the expected mounting of the humoral response. Additionally, day 14 was chosen as geese infected with WNV lineage 1 in Italy showed clinical signs in the third week after infection [28]. Day 21 was selected for the detection of overall serological response. To answer all these questions accurately, we implemented these different time points in this full-extent pathogenesis study for a detailed virological, serological, and pathohistological examination.

## 2. Materials and Methods

### 2.1. Geese

One-day-old geese from a commercial poultry farm were used for the animal experiment (Lower Saxony, Germany). They all tested negative for WNV, Usutu Virus, and Salmonellosis before infection.

### 2.2. Virus

A WNV lineage 2 isolate from an infected great grey owl from Germany in 2018 was used (GenBank accession no.: MH924836). After isolation on Vero B4 cells, it was passaged once on *Aedes albopictus* (C6/36) cells and once on Vero B4 cells (both obtained from Collection of Cell Lines in Veterinary Medicine, Friedrich-Loeffler-Institut, Germany). The virus stock was harvested after 4 days and kept at −70 °C in 500 µL aliquots. The concentration was titrated on Vero B4 cells and calculated as being of 10^7.5^ tissue culture infective dose 50 (TCID_50_) per mL [33].

### 2.3. Subcutaneous Injection and the Procedure for the Animal Trial

**Figure 1:**
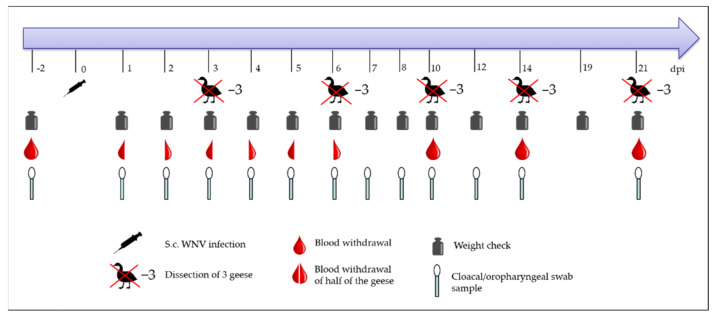
Planned time schedule of the animal experiment, including the sampling procedure over 21 dpi.

Fifteen approximately three-week-old geese were subcutaneously inoculated with 10^6^ TCID_50_ of WNV diluted in 500 µL minimum essential medium (MEM) by injecting 250 µL in each knee fold. Three geese were not inoculated and referred to as controls, and they were housed in a separate stable (Table 1). Afterwards, the infected geese were monitored daily by a veterinarian using a score sheet (which included daily recordings for behaviour, posture, respiration, plumage, appetite, and faeces, in addition to eye and nutritional condition recordings on sampling days only; Table S1) as well as by video camera. The geese were weighed regularly (2 days prior to infection, from day 1 to 10, daily; and 12, 14, 19, and 21 days post-infection (dpi)) to check for continuous gain in weight (Figure 1). When the geese scored over 15 points or lost more than 20% of their weight, they reached the humane endpoint and had to be euthanized via isoflurane inhalation followed by exsanguination from the carotid arteries (Table S2). Per planned necropsy time point (3, 6, 10, 14, and 21 dpi; 20 days for the uninfected geese, respectively), three geese were removed from the experiment under the same procedure of euthanasia. All of the geese were provided with water and food ad libitum. They had access to a small water basin for plumage care.

### 2.4. Sample Collection

Two days prior to infection, all geese were sampled to exclude flavivirus antigen or antibodies (Figure 1). Blood samples were taken from the Vena metatarsalis plantaris superficialis from days 1 to 6, 10, and 14 pi. For animal welfare reasons, the geese were split into two groups from 1 to 6 dpi and blood was withdrawn daily from alternate groups. The blood was centrifuged at 2500 rpm for the plasma and 3500 rpm for the serum for 10–15 min and the samples were frozen at −70 °C separately to the blood cruor.

Swab samples were taken with cotton swabs from the oropharynx and cloaca of all of the geese prior to infection, from day 1 to 10 pi daily and on days 12, 14, and 21 pi. The swab ends were put into screw-cap tubes filled with 2 mL MEM substituted with antimicrobials (gentamicin, amphotericin B, lincomycin, and enrofloxacin). After shaking at room temperature for 30 min at 50 rpm (Duomax 1030, Heidolph Instruments, Schwabach, Germany), the supernatant was decanted into 2 mL sterile tubes and stored at −70 °C.

Upon necropsy, various tissue samples were taken with separate tweezers and scissors per animal to avoid cross-contamination of samples. For virological examination, a pinhead-sized piece of each organ was stored in 2 mL sterile tubes with 500 µL MEM substituted with penicillin and streptomycin; another part was frozen at −70 °C without medium. For histopathological examination organs were also kept in 4% neutral buffered formalin.

### 2.5. Reverse Transcription Quantitative Real-Time PCR (RT-qPCR)

Pinhead-sized blood cruor was homogenized (2 min at 30 Hz; TissueLyser II, Qiagen, Hilden, Germany) in 600 µL RNeasy Lysis Buffer (RLT) (Qiagen) plus 6 µL ß-mercaptoethanol together with one 5 mm steel bead. Viral RNA was then extracted using the RNeasy Mini Kit (Qiagen), according to the manufacturer’s instructions.

The organ samples (brain, heart, kidney, liver, spleen, lung, bursa cloacalis, and feather pulp) were also homogenized with one 5 mm steel bead. For RNA extraction, only 100 µL of the homogenized organs and the swab samples were used in the BioSprint 96 (Qiagen) with the NucleoMag VET Kit (Macherey-Nagel, Düren, Germany). All of the RNA extracts were examined with the specific WNV RT-qPCR assay targeting the 5′ untranslated region (5′ UTR) for the simultaneous detection of lineage 1 and 2 strains [34]. For the quantification of viral RNA copies in each sample, a calibration curve of synthetic WNV RNA was run in parallel using 6-fold serial dilutions [34]. Additionally, the native virus was diluted, extracted, and used to estimate the TCID_50_/mL.

### 2.6. Virus Titration

Organ and blood cruor samples that tested positive in the RT-qPCR were additionally analysed by virus titration with an endpoint dilution assay on Vero B4 cells, and the virus titer was calculated using the Spearman–Kaerber algorithm [33].

### 2.7. Serology

Serum samples (from days 3–7, 10, 14, and 20/21 pi) were heat-inactivated (30 min at 56 °C) and analyzed by a virus neutralization test (VNT) as described by Seidowski et al. [35]. For this purpose, Vero B4 cells and the same German WNV strain as in the animal infection experiment were used. The neutralizing antibody titer (ND_50_) was determined as the reciprocal of the serum dilution that inhibited more than 50% of the cytopathic effects and calculated according to the Behrens–Kerber method [36]. Additionally, plasma samples (from days -2, 3–7, 10, 14, and 20/21 pi) were also examined in the competitive IgG enzyme-linked immunosorbent assay (competition ELISA) (IDvet, Grabels, France), according to the manufacturer’s instructions.

### 2.8. Histopathology and Immunohistochemistry

A tissue selection of muscle, plexus brachialis, liver, kidney, spleen, heart, lung, small and large intestine (including the cecal tonsil), and brain samples were stained with haematoxylin and eosin as well as by immunohistochemistry. The tissues were fixed in 4% neutral buffered formalin for at least 2 weeks before dehydration and embedding in paraffin. Sections (3 µm) were prepared and mounted on Superfrost plus slides (Menzel, Darmstadt, Germany). An in-house polyclonoal antibody (OM8) was used for the specific detection of WNV antigens. The pretreatment included rehydration, inhibition of endogenous peroxidase with 3% H_2_O_2_ (Merck, Darmstadt, Germany) in methanol for 30 min, a Proteinase K (Roche, Mannheim, Germany) treatment (with 4 µg/mL for 15 min at 37 °C), and a serum block directly before incubation with the antibody. The primary antibody was applied at a dilution of 1:1700 in goat serum and was incubated for 2 h at room temperature. Negative control sections were incubated with goat serum alone. For development, we used the EnVision reagent (Dako Diagnostics, Hamburg, Germany) and diaminobenzidine tetrahydrochloride, counterstained with Mayer’s haematoxylin.

### 2.9. Ethical Approval

The animal experiment was conducted under biosafety level 3 regulations and, with regard to animal welfare, following national and European legislation, in particular, directive 2010/63/EU. The experiment was approved by the State Office of Agriculture, Food Safety, and Fishery of the federal state of Mecklenburg–Western Pomerania, Germany (LALLF reference number: 7221.3-1-031/20; approved 15 June 2020). Throughout the experiment food and water was provided ad libitum, as well as a water source for cleaning and hay cobs for picking.

## 3. Results

### 3.1. Clinical Signs and Weight Gain

**Figure 2:**
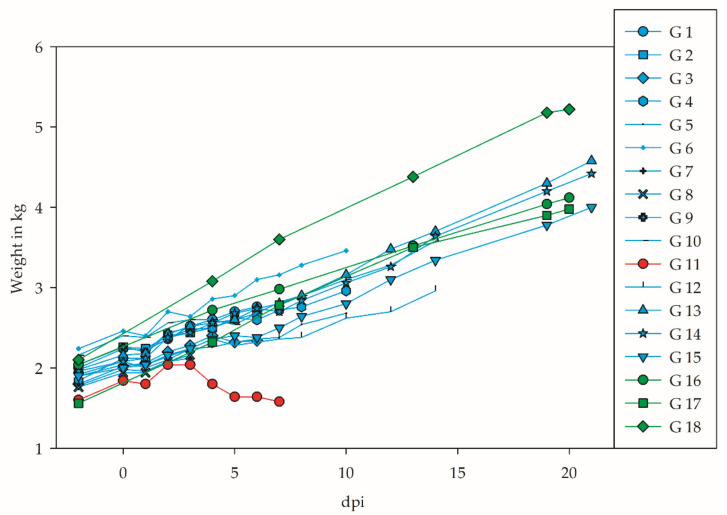
Weight gain of all geese throughout the experiment. Infected geese shown in blue, controls in green, and G 11 (severely diseased) in red.

Except for one goose, only minor, non-specific clinical signs (such as increased prostration and shivering) were observed from days 5 to 10 pi in 5 of the 15 infected geese. One goose (G 11), additionally, showed a staggering gait (5–6 dpi) as well as apathetic behavior, isolation from the group, and a retracted neck at 7 dpi (day of euthanasia). The same conspicuous goose (G 11) also did not gain any more weight after 3 dpi (Figure 2). All of the other infected and control geese showed steady weight gains.

### 3.2. Viremia and Virus Shedding

**Figure 3:**
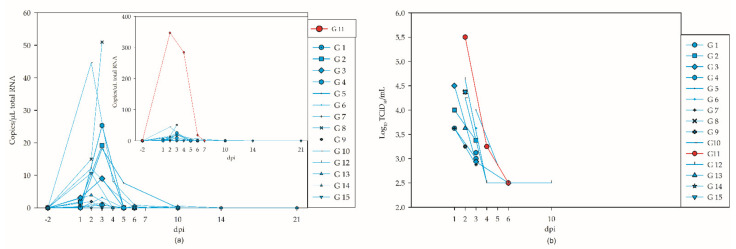
Viremia of the geese. The negative results of the controls (G 16–G 18) are not shown. (**a**) Results of RT-qPCR without G 11 (left) and for all WNV-inoculated geese (top right), using different y-scales. (**b**) Results of virus titration for RT-qPCR-positive samples.

All of the infected geese developed various degrees of viremia from day 1 (4/7) to 10 (1/8; days 8 and 9 not examined) (Figure 3a,b). As determined by RT-qPCR, the peak was reached at 2 dpi with 347 copies/µL total RNA for G 11 (Figure 3a, Table S3). The samples taken before the infection and all the samples of the controls tested negative in RT-qPCR tests. Regarding titration, serum from day 1 (4/8) until day 10 (1/8) contained viable virus, with quantities as high as 10^5.5^ TCID_50_/mL detected at 2 dpi (G 11) (Figure 3b).

**Figure 4:**
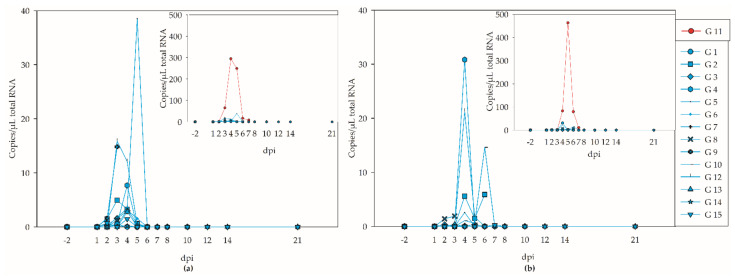
Viral shedding of the geese, without G 11 (left), and of all WNV-inoculated geese (top right), as estimated by RT-qPCR and using different y-scales. The negative results for the controls (G 16–G 18) are not shown. (**a**) Oropharyngeal shedding. (**b**) Cloacal shedding.

Viral shedding followed the viremia curve with a time delay, with shedding from days 2 (6/15) to 7 (only G 11) pi (Figure 4a,b; Table S4). The peak of the viral shedding in most of the geese was at 4 dpi (Figure 4a,b; Table S4). The highest viral load with 463 copies per microliter of total RNA was in the cloacal swab of goose G 11 at 4 dpi (Figure 4b, Table S4). Nevertheless, there were more positive oropharyngeal swabs (Figure 4a, Table S4) than cloacal swabs, and six of the infected geese never had a positive cloacal swab. The swab samples before the infection and all swabs of the controls at all time points tested negative by RT-q PCR.

### 3.3. Serology

**Figure 5:**
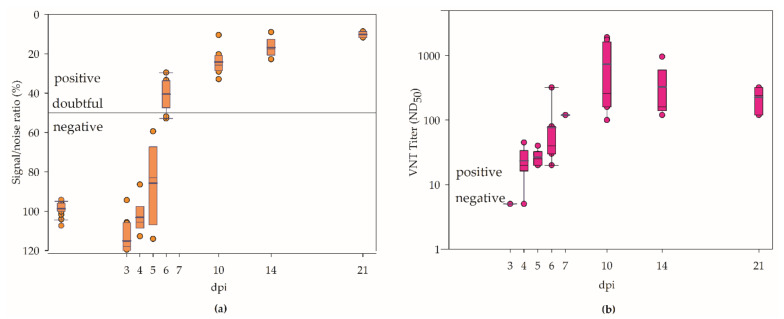
Seroconversion of all WNV-inoculated geese. Data are presented in a box-and-whisker plot, with the box including 50% of the values for each group and the dark-blue line in the middle of each group representing the median value. (**a**) Antibodies examined by a conventional competition IgG ELISA dyed in orange. (**b**) Antibodies examined by VNT dyed in pink.

All infected geese remaining in the experiment seroconverted at the latest 10 dpi in the IgG ELISA (Figure 5a). WNV-specific antibodies were detected even earlier in VNT, with titers reaching up to 1920 ND_50_ at 10 dpi (Figure 5b). The controls did not develop any IgG or WNV-specific antibodies. The apparent drop in the antibody titer in the VNT after 10 dpi (Figure 5b) can be attributed to the fact that birds with especially high antibody levels were removed 10 and 14 dpi due to a random selection of the animals for necropsy.

### 3.4. Tissue Tropism

**Figure 6:**
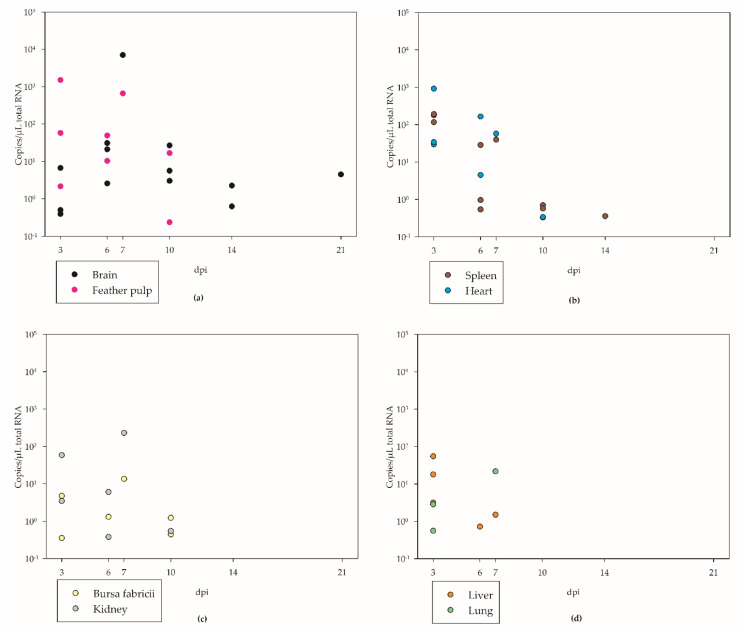
WN viral load in different organs of all WNV-inoculated geese as estimated by RT-qPCR (negative values are not shown) in: (**a**) brain and feather pulp; (**b**) spleen and heart; (**c**) bursa fabricii and kidney; and (**d**) liver and lung samples.

**Figure 7:**
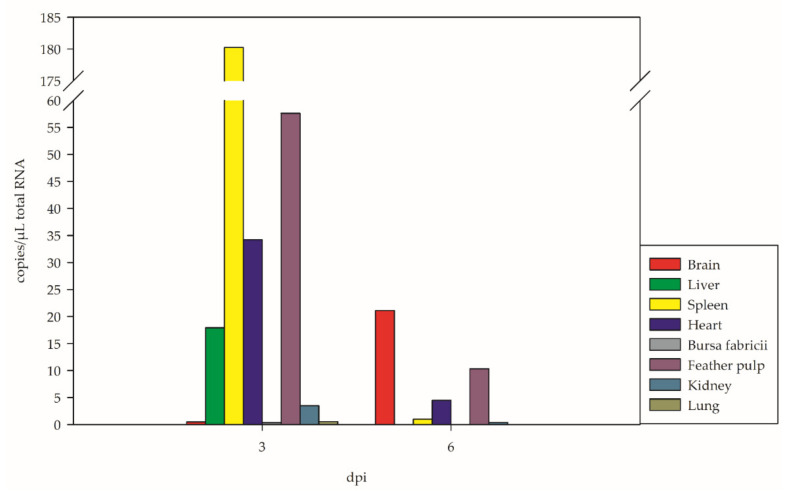
Viral distribution in different organs presented as the means of three WNV-inoculated geese each at 3 dpi and 6 dpi in copies/µL total RNA, as estimated by RT-qPCR.

The distribution of WNV in eight examined organs (brain, liver, spleen, heart, bursa fabricii, kidney, lung, and feather pulp) showed marked changes over time (Figure 6 and Figure 7, Table S5). It depends mainly on the time point of examination (and animal-specific patterns): at 3 dpi the virus was detected in all organs by RT-qPCR and was predominant in the feather pulp (up to 1510 copies/µL total RNA for G 8), followed by the heart (up to 921 copies/µL total RNA for G 8), and the spleen (up to 193 copies/µL total RNA for G 8) (Figure 6, Table S5). Additionally, according to virus titration (Table S6), viable virus was highest in the feather pulp (up to 10^6^ TCID_50_/mL organ suspension for G 8) and heart (up to 10^4.82^ TCID_50_/mL organ suspension for G 8), both at 3 dpi.

At 6 dpi, the viral loads were much lower and were highest in the heart (up to 165 copies/µL total RNA for G 3) (Figure 6, Table S5). Viable virus was only isolated from the brain (up to 10^2.25^ TCID_50_/mL organ suspension for G 3) and feather pulp (up to 10^1.86^ TCID_50_/mL organ suspension for G 2) (Table S6) of one goose, respectively.

Goose G 11, which was removed from the experiment at an early stage (7 dpi), showed very high viral loads in all of the examined organs, especially in the brain (up to 7000 copies/µL total RNA) via RT-qPCR (Figure 6, Table S5). Virus titration showed the highest load in the feather pulp (up to 10^5.36^ TCID_50_/mL organ suspension) and kidney (up to 10^5.13^ TCID_50_/mL organ suspension) (Table S6).

From days 10 and 14 pi, viral copies were determined in the brains of all of the examined geese (with copies up to 26 copies/µL total RNA) and in 4 out of 6 spleens (up to 0.7 copies/µL total RNA) (Figure 6, Table S5). Viable virus was only detectable in one brain (10^2.38^ TCID_50_/mL organ suspension for G 5) and one spleen (10^1.69^ TCID_50_/mL organ suspension for G 12) (Table S6). At 21 dpi, viral copies were only detected in one brain, and no viable virus was proven by virus titration (Table S6).

### 3.5. Gross Lesions, Histopathology, and Immunohistochemistry

No gross lesions typical for viral infection were visible. Only the clinically affected G 11 showed a reduced body condition as compared to all other animals; this was additionally associated with mild necrosis of the fatty tissues. Moreover, the spleen and bursa fabricii were very small in this animal, and the spleen had a fragile consistency.

**Figure 8:**
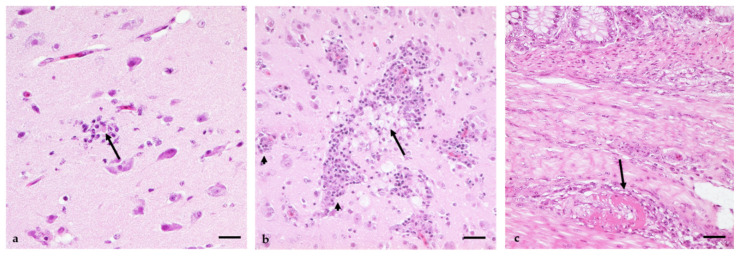
Histomorphological lesions induced by WNV infection in geese. (**a**) Small glial nodule with central neuronal necrosis in brain/cerebrum (arrow), G 5, 10 dpi. (**b**) Multifocal perivascular cuffs (arrowheads) in the brain/cerebrum. Clearly visible are the mild foci of encephalomalacia with gemistocytes clearing the cellular debris (arrow), G 11, 7 dpi. (**c**) Gut with distinct fibrinoid-necrotizing arteritis (arrow) in a medium size vessel of the tunica muscularis, G 11, 7 dpi. H&E, bars = 20 µm.

In histopathology (Table S7), all infected animals revealed a multifocal acute or subacute non-suppurative necrotising encephalitis of variable degrees, from weak (3/15) and mild (2/15) up to moderate (10/15). However, very weak signs of inflammation consisting of single glial nodules and satellitosis, as well as isolated weak perivascular lymphohistiocytic cuffs, were limited to the cerebrum (2/3) and cerebellum (1/3) of animals in the 3 dpi group. In contrast, all other geese (12/15) showed clear signs of encephalitis, which in most cases were evenly distributed throughout the brain, with the mesencephalon being the least affected area. The lesions were mainly multifocal perivascular lymphohistiocytic cuffs (13/15), in some cases with plasma cells, and were observed as having striking patterns, often with marked lymphocytolysis (8/13). Moreover, glial nodules (13/15), endothelial hypertrophy (7/15), satellitosis (6/15), astrogliosis (3/15), as well as necrosis of single Purkinje cells (2/15), were also seen. Only one animal showed signs of mild lymphohistiocytic meningitis. In most animals (12/15), only single-cell necrosis of scattered glial cells and neurons was visible (Figure 8a), but three geese revealed a mild encephalomalacia (3/15); this was most prominent in the clinically affected G 11 (Figure 8b).

Additional findings, most probably associated with WNV, were mild acute non-suppurative neuritis of peripheral nerves of the plexus brachialis (7/15), which were detectable in all groups. However, the additional ganglioneuritis, which was mainly detectable in the plexus myentericus (8/15), was confined to the earlier time points during the incubation period at 3 dpi (1/3) and 6–14 dpi (7/9) and was not seen at 21 dpi.

The spleen showed a variable appearance and depletion was detected in five animals (5/15); one of these animals (1/5) and another (1/10) also showed acute necrotizing splenitis. The clinically affected animal G 11 had the most prominent spleen lesions with distinct fibrinoid-necrotizing arteritis and depletion and distinct infiltration of tingible body macrophages. However, in most cases, follicles (10/15) with central lymphocytolysis were detectable.

Vasculitis was seen in six geese (6/15) at various locations, sometimes in more than one per animal. These locations included the spleen (3/6), heart (2/6), brain (2/6), and gut (1/6). In most cases, an acute non-suppurative arteritis was detectable, except for G 11, which showed a fibrinoid-necrotizing arteritis in the spleen and gut (Figure 8c).

A mild acute necrotizing myocarditis was only seen in four animals (4/15), whereas in six additional geese an unspecific multifocal mild interstitial lymphohistiocytic infiltration was seen. This entity was also seen in one of the control geese.

Accidental findings were most prominent in the liver, which showed subcapsular acute to subacute/chronic hemorrhages (10/15), in some cases associated with necrotic foci (7/15). However, similar hemorrhages were also seen in control animals and were interpreted as symptoms of fatty liver disease. An additional goose showed mild hepatocellular hydropic degeneration associated with acute hemorrhages and a chronic thrombophlebitis. Two geese also showed multifocal subacute-to-chronic pericholangitis. Three animals, including one control and G 11, revealed signs of enteritis induced by parasites. Tables S7 and S8 give an overview of the most important histomorphological and immunohistochemical results.

**Figure 9:**
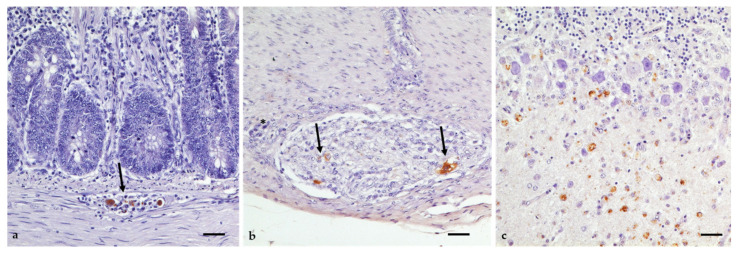
Immunohistochemical detection of WNV antigens in geese. (**a**) Plexus submucosus in the gut of G 1 (6 dpi) with viral antigen (arrow). (**b**) Plexus myentericus in the gut of G 11 (7 dpi). Viral antigens are distinctly seen in neurons and glial cells (arrows). Additionally, slight lymphohistiocytic infiltration can be seen (star). (**c**) Cerebellum/molecular layer of G 11 with a foci of altered neurons and glial cells accumulating viral antigens. Immunohistochemistry, pab (in-house) OM8; bars = 20 µm.

Viral antigen was detectable by immunohistochemistry in low amounts and only in individual animals (Figure 9, Table S8). The most prominent staining reaction was surprisingly found in the enteric nervous system (plexus submucosus and myentericus; Figure 9a,b) of G 2, G 3, and G 11, all from the 6/7 dpi group and in all cases associated with inflammatory infiltration. The mild reaction pattern was confined to neurons and glial cells of the multifocal plexus. In G 11, mild-to-moderate scattered neurons and glial cells in the molecular and granular layer of the cerebellum were found to be positive, too (Figure 9c).

## 4. Discussion

This study is the first to examine the pathogenesis of a German WNV strain (lineage 2, from 2018) in vivo under experimental conditions. Moreover, we focused on farm birds, aiming to clarify the role of these animals in the spread of WNV over the time course of the disease. A similar study had already been performed with geese, albeit the birds were infected with a WNV lineage 1 isolate from Italy [28]. At the time of implementation, WNV had not been detected in Germany and it was suspected that WNV lineage 1 strains would be introduced into the country from the south [28]. In contrast to expectations, WNV lineage 2 was introduced to Germany in the eastern part of the country (Saxony-Anhalt) in 2018 from the Czech Republic [21].

In the past, WNV lineage 1 (clade 1a) was associated with encephalitis and fatal cases in geese in Israel [37], USA [6], Canada [8], and Hungary [12]. In Israel, a high mortality rate of up to 40% was observed in goose flocks [37], with simultaneous detection of clinical symptoms in goose keepers [38], whereas in a natural outbreak among geese in Canada, the mortality rate was only 25% [8]. This strikingly higher susceptibility of domestic geese to WNV in Israel had not been reported previously and may indicate that a change in the WNV genome possibly resulted in geese becoming incidental hosts (similar to what occurred in Bucharest in 1996) [39]. Also recently, two geese in Israel succumbed to infection with WNV lineage 1, with and without displaying neurological signs, respectively [40]. Therefore, it is important to be able to assess the pathogenicity of German WNV isolates and their associated risks to farm poultry, birds, and humans.

In this study, we therefore infected young geese with a German WNV lineage 2 isolate and regularly collected blood, swab, and organ samples. In essence, all of the inoculated geese were susceptible to WNV, as shown by positive results for RT-qPCR tests of blood as well as tissue and swab samples performed at different time points during the incubation period. Moreover, a distinct time-dependent detection of viral antigen and virus was obvious; at 3 dpi, all animals were positive, but at 21 dpi only one animal revealed WNV antigens in the brain. Furthermore, all geese showed clear seroconversion, starting from day 5 and detectable until the end of the experiment (day 21), and showed histomorphological lesions in different tissues as is typical for a viral infection.

Interestingly, one goose (G 11) developed very high viremia levels of 10^5.5^ TCID_50_/mL serum (Table S3), corresponding to approximately 10^5.3^ PFU/mL serum at 2 dpi. According to the literature, a titer higher than 10^5^ PFU/mL in the blood of a host (proven for American *Culex* spp.) [2] or 10^6^ PFU/mL, respectively [41], is required to infect mosquitoes by blood meal feeding. It is therefore questionable whether the WNV titer in the blood of G 11 would be sufficient to sustain the natural WNV transmission cycle. This animal was also the only goose showing distinct clinical signs and had to be euthanized for animal welfare reasons.

During the necropsies, we also pulled feathers from the dead animals for virological examination of the feather pulps. With this, we wanted to investigate a possible route of horizontal virus transmission. The feather pulps were frequently infected in 9/15 geese up to 14 dpi, as detected by RT-qPCR, containing up to 10^6.1^ TCID_50_/mL (G 8, 3 dpi), as proven by virus titration (Table S6). Thus, the virus was detectable in the feather pulps for a longer duration than in the blood in both our study and in red-legged partridges (*Alectoris rufa*) infected with a WNV lineage 1 strain [42]. Therefore, our results not only support direct horizontal transmission cycles via pulp by feather pecking but also indicate an alternative method for collecting and analyzing feather pulps for the monitoring of WNV in live/succumbed (wild/zoo) birds. This is also in agreement with data on carcasses of American crows (*Corvus brachyrhynchos*) and blue jays (*Cyanocitta cristata*), where feather pulps constituted the tissue sample that most frequently tested positive for WNV [43]. The precise reason as to why WNV accumulates in the feather pulps is still unknown. Feather pecking, however, is a common problem in conventional farms and is even more frequent among sick and defenseless individuals in the flock. Additionally, in this study, feather pecking was observed during stress (transport) or boredom (controls).

Normally, it is not necessary for an arbovirus to be shed via secrets or feces as their natural cycle involves insects as vectors. Nevertheless, Langevin et al. [44] were the first to describe shedding of WNV by infected chickens. Furthermore, Swayne et al. [27] proved direct transmission of WNV lineage 1 (NY 99) between infected and non-infected geese even though the viral titers in the oropharyngeal swabs were fairly low (≤10^2.5^ TCID_50_/mL swab media). In a further study, two in-contact geese housed with WNV-infected geese (lineage 1 from Israel; IS-98-ST1) even died, although only three cloacal and one tracheal swab were WNV-positive [26]. However, also in Canadian geese infected with WNV lineage 1 NY 99, only very low titers were found in oral swabs [2]. In our study, the absolute viral load was higher in the cloacal swab from G 11 than in the oral swab at 5 dpi, as in any other blood sample. However, the cloacal swabs were positive less often and there was no cloacal shedding proven in 6 out of 15 individuals over the whole time of the trial. Despite the high copy numbers in the swabs analyzed by RT-qPCR, we unfortunately could not find viable virus by virus titration. In other studies [29,45], also, no viable virus was detected in the swabs despite high genome copy numbers sometimes being found by PCR. Taken together, since the viral loads in most of the swabs examined here were relatively low, the possibility of direct horizontal viral transmission by feather pecking becomes more likely.

This study provides insights into the tissue distribution of WNV over time. In the early pathogenesis (3 dpi), the virus dominated in the spleen but could be confirmed in all examined organs. At day 6 pi, viral loads were generally lower, except for constant values in the feather pulps and a strong increase in the brain. Subsequently, neurotropism dominated (10–14 dpi). Towards the end of the experiment (21 dpi), viral clearance occurred, with one exception, G 15, which had only low viral copies in the brain. This is in contrast to results for house sparrows (*Passer domesticus*), house finches (*Carpodacus mexicanus*), and Western scrub jays (*Aphelocoma californica*), with a persistence of WNV RNA in kidneys and spleens over several months [46]. This was probably due to the higher susceptibility of these bird species to WNV such that they developed higher viral loads directly after infection.

This time-dependent pattern of virus infection is also reflected in the histomorphological lesions (Figure 8) outside of the brain, which were most prominent 3–14 dpi. For example, in the from days 3 to 10 pi there was a depletion of lymphocytes and necrotizing splenitis in individual animals. Signs of vasculitis were also seen at different locations in some of the geese at that time. However, in the brain, clearly visible signs of encephalitis were not seen before 6 dpi but were distinctly seen until 21 dpi, even after the clearance of viral antigens. Moreover, we have indications that the enteric nervous system also plays a key role in the early replication cycle of the virus, with inflammatory alterations (lymphohistiocytic ganglioneuritis) shown in eight animals, already starting at 3 dpi and lasting until 14 dpi. In three cases, viral antigens were also detectable in neurons and glial cells of the plexus, indicating a high amount of viral load at this site (Figure 9a,b). These findings are consistent with previous studies of WNV in birds: the spleen is infected first, with the virus replicating in macrophages, followed by dissemination to other organs and finally reaching the CNS by overcoming the blood–brain barrier [47,48].

As already concluded by others, clinical disease occurs simultaneously with the invasion of WNV in the CNS or other major organs, including the liver, spleen, kidneys, and heart [49]. If host defense fails, death occurs between 24 h and 4 days after the onset of clinical signs [48]. This was probably the case with G 11: after the peak of viremia at 2 dpi, clinical signs appeared not prior to 5 dpi, and the situation eventually worsened dramatically at 7 dpi, despite increasing antibody titers. This is consistent with the course of viremia in geese, with clinical signs often appearing 5 dpi [32]. It is reflected by the extremely high viral loads in the brain detected by RT-qPCR, virus titration, and IHC, as well as the most prominent histomorphological lesions characteristic of viral infection, including a necrotizing encephalitis, fibrinoid-necrotizing arteritis in the spleen and gut, as well as depletion of the spleen. However, besides some incidental findings, i.e., a mild parasitic infection which was seen in other animals as well, no histomorphological signs of an additional severe entity affecting the immune status of this animal were observed. Furthermore, all 15 geese plus the 3 controls were acquired as day-old hatchlings from the same flock and were housed under controlled experimental settings. Therefore, individual genetic predisposition may have played a role in this animal’s high susceptibility to WNV, as has been found by others [50,51].

Histopathological lesions of most of the geese were characteristic of viral infection and in accordance with previous reports [29,48,52]. The main finding was an acute necrotizing encephalitis, which was seen in almost all geese, the first signs already starting 3 dpi. Three individuals even showed small foci of encephalomalacia at 14 dpi, which is usually only seen in highly susceptible birds of prey [53], showing the susceptibility of this species to the disease. An additional characteristic finding for WNV infection was the involvement of vessels, in six cases with a non-suppurative arteritis, but in G 11 even with a distinct fibrinoid-necrotizing arteritis. To our surprise, a distinct lymphohistiocytic ganglioneuritis was obvious in several birds at early stages of the disease—a lesion which has been described before in WNV-infected geese [27] and is typically associated with avian Borna virus infection, as reviewed by Staeheli et al. [54].

Unspecific signs of viral infection were splenic lymphoid depletion and necrosis in single animals. Splenic lymphoid depletion has up until now only been described for northern goshawks (*Accipiter gentilis*) infected with WNV lineage 2 [55] and not WNV lineage 1 [48]. Additionally, the lymphohistiocytic neuritis of peripheral nerves detectable in several geese can be seen in several entities [29,56,57] and has to be regarded with care. However, in particular, the minor involvement of the heart, with only mild lymphohistiocytic necrotizing myocarditis observed in the geese presented here, came as a surprise, as in several species the heart is, besides the brain, the most affected site in WNV infection in birds of prey [58,59]. However, this finding is in accordance with previous studies in geese [28,49]. Taken together, a combination of necrotizing encephalitis and vasculitis, mixed with lymphohistiocytic ganglioneuritis in the ENS and additional unspecific histopathological signs of viral infection, is clearly indicative of WNV infection in geese. However, it should be noted that diagnosis by immunohistochemistry is difficult in this species, as only three of our geese revealed detectable viral antigens by this method (Tables S7 and S8). This is in accordance with the viral load seen in RT-qPCR (Figure 6, Table S5), showing positive IHC staining only with CT values below 20. Furthermore, in other studies, IHC was positive only at high viral loads detected by RT-qPCR and at early time points pi because of the lower sensitivity compared to RT-qPCR [42,58,59]. This lower sensitivity could be due to the fact that the antibody of the IHC detects a different target than the RT-qPCR or that only many small amounts of antigen are present in the tissue.

For susceptibility to WNV, age also plays a major role as a key factor in the immune system. In an earlier study from Banet-Noach et al. [26], geese aged 3–8 weeks were most susceptible, even though older geese up to 12 weeks of age were also affected. Furthermore, in the outbreak in domestic geese in Canada, WNV most severely affected the 6-week-old cohort, with a mortality of 25%, while the cohorts of 15-month-old and 5-year-old geese showed only seroconversion without clinical disease [8]. A similar observation was made during the WNV outbreak in geese in Israel [38]. As already proven for other viruses, the course of infection in the host can also be intensified by the means of virus transmission via mosquito inoculation as compared to subcutaneous inoculation of animals [28,60].

In this study, similar to the infection with WNV lineage 1 Italy in geese, viremia, virus excretion (excluding G 11), as well as viral loads and lesions in the brain at 21 dpi were comparable. The higher viral loads in the organs in our study correspond to earlier time points of examination and therefore with the progression of the infection. However, it appears that the clinical signs in geese infected with the German lineage 2 strain were noted earlier and as being more severe than in geese with an infection with WNV lineage 1 Italy [28]. One explanation for this could be the fact that the viremia of the geese with WNV lineage 1 Italy was slightly lower and therefore the crossing of the blood–brain barrier was delayed. As the crossing of the blood–brain barrier is dependent on the viremia [48], viremia is a key factor in the pathogenesis of the neurotropic flaviviruses, resulting in severe clinical outcomes [61].

In other studies on highly susceptible large falcons (*Falco* spp.) and magpies (*Pica pica*), there were also no significant differences (in clinical signs, viremia, excretion, pathological lesions, and serology) between infections with WNV lineages 1 and 2, as, for example, can be seen when comparing WNV lineage 1 NY 99 with WNV lineage 2 Austria 2009 [29] and lineage 2 SRB-Novi Sad/12 strain from Serbia [62], respectively. In jackdaws (*Corvus monedula*) and carrion crows (*Corvus corone*), also, the pathogeneses of lineage 1 and 2 strains were comparable except for one less pathogenic strain from Italy (FIN) [30,63]. This is contrary to results for red-legged partridges, as infection with WNV lineage 1 Italy was more pathogenic than infection with WNV lineage 1 Israel 1998 or WNV lineage 2 Austria 2008 [31]. When comparing the virulence of different WNV variants, focus should not only be on the different lineages but also on the strains themselves. The closely related WNV lineage 1 strains from the outbreaks in Israel 1997–2000, USA 1999, and Hungary 2003 were neurovirulent and often fatal in geese [6,38,64]. By contrast, the Italian WNV strains also belonging to lineage 1 only caused mild clinical signs starting two weeks pi [28]. Regarding seroconversion, we could prove that it already began 4 dpi (Figure 5); this was not examined in detail in the other study [28].

The main criteria for an arbovirus sentinel bird include susceptibility to infection, development of detectable antibodies, and survival of an infection. Otherwise, they should not participate in the natural transmission cycle nor spread the virus directly [44]. In the WNV outbreak in New York in 1999, pigeons were found to have high levels of neutralizing antibodies [65,66]. Therefore, they are used as nonmigratory free-ranging sentinel animals for the monitoring of WNV among others in South Africa [67], Spain, [68], Greece [69], and Georgia, USA [70]. The use of chickens as sentinels has been carried out, for example, in South Africa [71], Australia [72,73], Romania [74], Greece [75], and in several states in the USA [66,76]. In the USA, however, this was only partially instructive, since seroconversion of the chickens was only observed after humans had become ill [77]. In contrast, pigeons were a useful early warning system for human infections in Greece [69]. Hence, domestic geese should be kept in mind as possible captive sentinels for the monitoring of WNV antibodies, as the majority of geese in this study did not display clinical signs upon infection and they all clearly seroconverted in a timely manner. Compared to chickens, geese react stronger and sooner to a WNV infection. In addition, domesticated geese are kept in human care and their blood collection is simple, both of them characterizing geese as feasible sentinels. Furthermore, they are kept outdoors in Germany in the summer, in line with the WNV season. Samples can also be taken easily in the fall during slaughter as a contribution to the monitoring of zoonotic diseases. However, it should be noted that individual geese can act as amplifying hosts, as shown by G 11. Especially after a mosquito-borne infection, viremia may be even higher and therefore transmission may occur. Furthermore, if geese are younger, with immature immune systems, severe infections are conceivable.

In addition to the findings of this study, the current epidemiological situation in Germany should be kept in mind. Previous infection with the closely related Usutu virus may also influence immune response to WNV infection. Further studies are needed to assess this risk for animal and human health.

## 5. Conclusions

Taken together, the interplay between the infecting virus strain and the host factors (other diseases, hormonal factors, stress, and age) is important for the pathogenesis of WNV. According to our pathogenesis study, there is only a very low risk of domestic geese becoming severely infected with WNV and acting as amplification hosts. However, free-ranging geese can serve as useful sentinel animals, especially in monitoring the spread of WNV and other arboviruses to new areas. On the other hand, there are still some unanswered questions regarding the neuropathology of WNV disease, e.g., how long the infection lasts and how the course of the inflammation is characterized.

## Figures and Tables

**Table 1:** Number of geese and necropsy time points.

Goose Number	Virus	Date of Euthanasia
G 1–G 3	WNV-2/s.c.	6 dpi
G 4–G 6	WNV-2/s.c.	10 dpi
G 7–G 9	WNV-2/s.c.	3 dpi
G 10, G 12	WNV-2/s.c.	14 dpi
G 11	WNV-2/s.c.	7 dpi ^1^
G 13–G 15	WNV-2/s.c.	21 dpi
G 16–G 18	No virus/control	20 dpi

^1^ This animal was taken out of the experiment unplanned and prematurely. s.c.: subcutaneous.

## Data Availability

The data that support the findings of this study are available in the main manuscript and in the Supplementary Materials for this article.

## Supplementary Materials

The following supporting information can be downloaded at: https://www.mdpi.com/article/10.3390/v14061319/s1, Table S1: Score sheet used for assessment of the health status of the animals; Table S2: Clinical scores of geese (G) infected with WNV via subcutaneous injection; Table S3: Results of RT-qPCR of blood cruor of all infected geese, indicated as Ct values and copies/µL total RNA; Table S4: Results of RT-qPCR of the cloacal swab samples of all infected geese, indicated as Ct values and copies/µL total RNA; Table S5: Results of RT-qPCR of organ homogenates of all infected geese, indicated as Ct values, copies/µL total RNA, and copies/mg original organ; Table S6: Results of titration of RT-qPCR-positive organ homogenates in TCID50/mL; Table S7: Histomorphological alteration of WNV-infected geese and controls; Table S8: Distribution of histomorphological alteration in the central nervous systems of WNV-infected geese and controls.
